# Serum mAST/ALT ratio had high predictive value for adverse outcome of severe fever with thrombocytopenia syndrome with severe condition

**DOI:** 10.1186/s12879-023-08121-2

**Published:** 2023-03-17

**Authors:** Li Wang, Youde Liu, Renliang Qu, Zhiqiang Zou

**Affiliations:** 1 Present Address: Clinical Laboratory, Qishan Hospital of Yantai, 62 Huanshan Road, Zhifu District, 264001 Yantai, Shandong The People’s Republic of China; 2 Present Address: Infectious Disease Department, Qishan Hospital of Yantai, 62 Huanshan Road, Zhifu District, 264001 Yantai, Shandong The People’s Republic of China

**Keywords:** Severe fever with thrombocytopenia syndrome, AST/ALT ratio, cAST/ALT ratio, mAST/ALT ratio, Risk factor

## Abstract

**Background:**

Severe fever with thrombocytopenia syndrome (SFTS) usually demonstrates multi-organ injury with a high mortality rate. This study aimed to investigate associations of serum aspartate/alanine aminotransferase (AST)/ALT, cytosolic AST (cAST)/ALT and mitochondrial AST (mAST)/ALT ratios with the prognosis of SFTS patients.

**Methods:**

A total of 355 confirmed SFTS patients were included. Clinical and laboratory data were compared between survivors and nonsurvivors. Logistic regression analysis was used to assess the independent risk factors for fatality in all patients and those admitted to the intensive care unit (ICU). The predictive values of the risk factors and constructed risk models were evaluated.

**Results:**

Mean age and biochemical parameters were significantly greater in nonsurvivors than in survivors. In ICU patients, the three ratios, high-sensitivity troponin I (hsTnI), creatine kinase (CK), lactate dehydrogenase (LDH) and α-hydroxybutyrate dehydrogenase (α-HBDH) were elevated markedly in nonsurvivors than in survivors. Multivariate logistic regression analysis showed that age, three ratios and α-HBDH were independent risk factors for mortality in all patients. Only the three ratios were independent risk factors for death in ICU patients. Risk Models (M1, M2 and M3) and simplified models (sMs) containing the three ratios respectively had comparatively high predictive values for fatality in all patients with area under ROC curves (AUCs) > 0.85. In ICU patients, mAST/ALT ratio had the highest predictive value, sensitivity and odds ratio (OR) for mortality among three ratios.

**Conclusion:**

AST/ALT, cAST/ALT and mAST/ALT ratios were associated with unfavorable clinical outcome of SFTS. The prognostic value of mAST/ALT ratio was higher in severe cases.

## Introduction

Severe fever with thrombocytopenia syndrome (SFTS) is an emerging hemorrhagic fever caused by a tick-borne banyangvirus and is associated with high fatality. SFTS virus (SFTSV) infection and epidemic regions are increasing annually worldwide and become a serious public health concerns globally, especially in East Asia [1]. The pathogenesis of lethal SFTSV infection in humans is not fully understood. Currently, no specific therapies and vaccines are available for SFTS. It is of utmost importance to make a precise assessment of the condition severity and predicts the progression in the early stage of treatment. SFTS is sepsis-like condition that the host response overwhelms infection leading to shock and multiple organ dysfunction [2]. For example, liver function abnormality was associated with increased risk of SFTS mortality [3], and fulminant myocarditis was reported as a complication of SFTS [4]. Mitochondria are key organelles of cellular energy metabolism which determines cell life and death [5]. Ultrastructural damage and mitochondrial dysfunction are reported in sepsis [6]. Mitochondrial DNA as the mitochondrial damageassociated molecular patterns (mtDAMPs) plays a major role in the development of sepsis [7]. MtDAMPs was reported to be involved in NLRP3 inflammasome activation in SFTS [8].

Aspartate aminotransferase (AST) is an organ-nonspecific enzyme whose serum elevation was observed in many tissue damages of the human body [9, 10]. The two isoforms of AST: cytosolic AST (cAST) and mitochondrial AST (mAST) are important components in the mitochondrial malate-aspartate shuttle (MAS) in cells [11]. As a major metabolic pathway, MAS was involved in many physiological processes, such as gluconeogenesis in the liver and kidney, glyceroneogenesis in the fatty tissue, and synthesis of many neurotransmitters, energy metabolism in microglia, and neuro-glial pathway in the brain [10, 12]. MAS abnormality was associated with various pathological processes. Mitochondrial enzymes are released into the blood only after severe injury of the cells, probably necrosis occurs, and the cumulative activity of mAST in the blood might reflect the extent of necrosis better than that of cAST in injured organs [13].

Previous studies showed that serum mAST was the biomarker of liver and myocardial necrosis [14, 15]. Therefore, assay of the mAST and cAST elevation accurately is necessary to assess cell injury and organ function. AST and AST/alanine aminotransferase (ALT) ratio have been reported as risk factors for adverse clinical outcomes of various diseases including SFTS [16]. Whereas, changes of serum mAST and cAST levels and their associations with muti-organ dysfunction, the disease severity and prognosis in SFTS had not been studied.

In this study, we aimed to estimate the serum level of cAST, mAST, cAST/AST and mAST/ALT ratios in the prediction of clinical outcome of SFTS patients with different severity and compare their efficacy with those of AST and AST/ALT ratio.

## Patients and methods

### Patients’ enrollment and data Collection

A total of 355 confirmed SFTS patients admitted in our hospital from April 1, 2020, to November 15, 2022 were included. SFTSV infections were diagnosed according to the results of real-time reverse transcriptase-polymerase chain reaction (RT-PCR) in peripheral blood samples. Demographic, clinical and laboratory data were obtained from individual medical records. Serum biochemical parameters of liver and cardiac injury were tested by Beckman AU5800 automatic biochemical analyzer. Level of cAST was calculated as AST-mAST. AST elevation was defined as ≥ 2 × upper limit of normal (ULN) (i.e., 80 U/L). And mAST elevation was defined as ≥ 2 × ULN (i.e., 36 U/L). Some of the mAST results were absent in patients with mild conditions.

### Ethics, consent and permissions

This study was approved by the ethics committee of Qishan hospital of Yantai, Shandong (Ethics number 202201), China and was carried out according to the Helsinki II Declaration.

### Statistical analysis

Continuous variables were expressed as the mean ± standard deviation (SD) for normal distribution or median (interquartile range) for skew distribution, and categorical variables were described as a frequency. The student *t* test or Mann–Whitney *U* test was used for comparing value difference between two groups. Correlation analysis was done using paired Spearman’s correlation analysis. Univariate and multivariate logistic regression analysis were used to assess independent risk factors for mortality. Chi-square was employed to determine difference between categorical variables of the groups. Receiver operating curve (ROC) analysis was used for the calculation of optimal cut-off value for risk factor and risk models. Risk models contain different independent risk factors were established. Area under ROCs (AUCs) with the highest Youden index was used to determine the predictive efficacy of the risk factors and the risk models. Log-rank test of the Kaplan Meier survival analysis was utilized to calculate the cumulative risk of parameters for mortality. SPSS software (version 26.0, IBM, Armonk, NY, USA) and MedCalc software were used for statistical analysis and *p* values < 0.05 were considered significant.

## Results

### Admission demographics and biochemical parameters of SFTS Patients

In 355 cases, 77 (21.7%) were admitted to ICU, 66 (18.1%) died. Mean age, and all of the biochemical parameters included were elevated markedly in nonsurvivors than in nonsurvivors in all patients. In patients admitted to ICU, serum levels of the three ratios, high-sensitivity troponin I (hsTnI), creatine kinase (CK), lactate dehydrogenase (LDH) and α-hydroxybutyrate dehydrogenase (α-HBDH) were elevated markedly in nonsurvivors than in survivors. The hospital stay was shorter in death group than in survival group. Females had a higher survival rate for all patients. Gender distribution had no difference in the two groups of ICU patients. Data are included in Tables 1 and 2.

**Table 1:** Comparison of demographic and peak biochemical data within a week of admission in survivors and non-survivors [mean ± SD or median (Q_25 −_ Q_75_)].

parameters	survivors	non-survivors	*P-*value
N (%)	289 (81.4)	66 (18.6)	-
Age (year)	65.2 ± 11.1	71.7 ± 8.7	< 0.001
Gender (M/F)	113/176 (39.1/60.1)	36/30(54.5/45.5)	0.02
Hospital stay (days)	11.6 ± 7.1	5.7 ± 4.4	< 0.001
ALT (U/L)	71.7 (44.4, 135)	122.9 (57.3, 224.8)	< 0.001
AST (U/L)	136 (74.2, 266.2)	371.3 (209.0, 750.7)	< 0.001
AST/ALT ratio	1.94 (1.4, 2.56)	3.52 (2.52, 4.54)	< 0.001
GGT (U/L)	31 (18, 58)	52 (23, 139.3)	< 0.001
ALP (U/L)	64 (51, 80)	78 (58.2, 158.5)	< 0.001
mAST (U/L)	34.3 (19.4, 68.7)	92.7 (55.2, 155.7)	< 0.001
mAST/AST ratio	0.242 (0.210, 0.258)	0.254 (0.245, 0.26)	< 0.001
mAST/ALT ratio	0.49 (0.35, 0.63)	0.77 (0.59, 1.02)	< 0.001
cAST (U/L)	101.4 (55.9, 194.8)	278.6 (153.8, 594.4)	< 0.001
cAST/ALT ratio	1.45 (1.05, 1.90)	2.72 (1.97, 3.45)	< 0.001
hsTnI (pg/ml)	50 (26, 107)	253.5 (75.5, 1539.5)	< 0.001
CK (U/L)	333 (125, 906)	1035 (435.5, 2597.3)	< 0.001
CK-MB (ng/ml)	3.63 (2.25, 7.14)	7.50 (4.39, 17.79)	< 0.001
α-HBDH (U/L)	351.0 (249.0, 537.8)	846.0 (552.4, 1439.3)	< 0.001
LDH (U/L)	538 (375, 846)	1422.5 (869.3, 2567.5)	< 0.001

ALT: alanine aminotransferase; AST: aspartate aminotransferase; mAST: mitochondrial AST; cAST: cytosolic AST; ALP: alkaline phosphatase; GGT: gamma-glutamyltransferase; hsTnI: high-sensitivity troponin I; CK: creatine kinase; CK-MB: CK heart-type isoenzyme; α-HBDH: α-hydroxybutyrate dehydrogenase; LDH: lactate dehydrogenase

**Table 2:** Comparison of demographic and peak biochemical data within a week of admission in survivors and non-survivors enrolled in ICU [mean ± SD or median (Q_25 −_ Q_75_)].

parameters	survivors	non-survivors	*P-*value
N (%)	44 (57.1)	33(42.9)	-
Age (year)	69.6 ± 6.5	71 ± 7.5	0.359
Gender (M/F)	17/27 (38.6/61.4)	18/15(54.5/45.5)	> 0.05
Hospital stay (days)	14.9 ± 12.5	7.5 ± 11.1	< 0.001
ALT (U/L)	120.6 (49.7, 181.3)	125.3 (61.5, 202.6)	> 0.05
AST (U/L)	204.3 (110.5, 494.6)	383.8 (2030.5, 745.5)	> 0.05
AST/ALT ratio	2.57 (1.94, 3.25)	3.83 (2.53, 4.77)	0.001
GGT (U/L)	44.5 (23, 144.3)	55 (26, 176.1)	> 0.05
ALP (U/L)	69.8 (53.3, 188.6)	99.0 (57.4, 158.5)	> 0.05
mAST (U/L)	54.0 (28.9, 120.8)	93.6 (59.1, 159.4)	0.005
mAST/AST ratio	0.25 (0.21, 0.26)	0.25 (0.23, 0.26)	> 0.05
mAST/ALT ratio	0.58 (0.45, 0.78)	0.81 (0.63, 1.15)	< 0.001
cAST (U/L)	153.9 (82.4, 376.2)	292.0 (171.6, 592.7)	0.012
cAST/ALT ratio	1.97 (1.43, 2.47)	3.09 (1.80, 3.57)	0.001
hsTnI (pg/ml)	117.5 (50, 280.6)	261.9 (90.0, 1914.5)	0.007
CK (U/L)	610 (270.5, 1292.8)	925 (474, 2438)	0.033
CK-MB (ng/ml)	6.59 (3.34, 14.42)	6.43 (4.05, 15.02)	> 0.05
α-HBDH (U/L)	527.8 (383.1, 864.2)	1093.9 (614.3, 1068.5)	0.002
LDH (U/L)	855.5 (521.5, 1543.3)	1750 (1095.5, 2792.5)	0.002

ALT: alanine aminotransferase; AST: aspartate aminotransferase; mAST: mitochondrial AST; cAST: cytosolic AST; ALP: alkaline phosphatase; GGT: gamma-glutamyltransferase; hsTnI: high-sensitivity troponin I; CK: creatine kinase; CK-MB: CK heart-type isoenzyme; α-HBDH: α-hydroxybutyrate dehydrogenase; LDH: lactate dehydrogenase

### Correlation analysis of parameters

Spearman’s correlation analysis showed that AST had a very strong correlation with mAST (r = 0.993, *p* < 0.001) and cAST (r = 0.999, *p* < 0.001). ASLT/ALT ratio also had a strong correlation with mAST/ALT ratio (r = 0.995, *p* < 0.001) and cAST/ALT ratio (r = 0.997, *p* < 0.001). Levels of CK, CK heart-type isoenzyme (CK-MB), hsTnI, LDH and α-HBDH had significant correlations with each other and other parameters (*p* < 0.001) except for age.

### Independent risk factors for adverse prognosis of SFTS

Univariate regression analysis indicated that age and all the biochemical parameters were independent risk factors for death in all patients. Only AST, mAST and cAST, and AST/ALT, mAST/ALT and cAST/ALT ratios were independent risk factors for death in patients admitted to ICU. Considering the multicollinearity among the parameters, AST and AST/ALT ratio, mAST and mAST/ALT ratio, and cAST and cAST/ALT ratio were included into multivariate regression analysis with other independent risk factors respectively. Results showed that age, α-HBDH, AST/ALT ratio, mAST/ALT ratio, and cAST/ALT ratio were independent risk factors for mortality in all patients. The three ratios were the only independent risk factors for death in patients admitted to ICU. And the odds ratio (OR) of mAST/ALT ratio was the highest among those of the independent risk factors. Results are demonstrated in Table 3.

**Table 3:** Independent risk factors obtained by multivariate logistic regression analyses for mortality during hospitalization in all SFTS patients and patients admitted to ICU

parameters	Multivariate regression			Multivariate regression			Multivariate regression		
	B	OR (95% CI)	*P-*value	B	OR (95% CI)	*P-*value	B	OR (95% CI)	*P*-value
For all patients									
Age (year)	0.092	1.096 (1.049,1.146)	< 0.001	0.088	1.092 (1.045,1.141)	< 0.001	0.094	1.099 (1.051,1.149)	< 0.001
AST/ALT ratio	0.71	2.034 (1.422, 2.91)	< 0.001						
mAST/ALT ratio				2.525	12.49 (3.252,47.927)	< 0.001			
cAST/ALT ratio							0.921	2.513 (1.582, 3.991)	< 0.001
α-HBDH (U/L)	0.003	1.003 (1.002, 1.004)	< 0.001	0.003	1.003(1.002, 1.005)	< 0.001	0.003	1.003 (1.002, 1.004)	< 0.001
For ICU patients									
AST/ALT ratio	0.676	1.967 (1.297, 2.983)	0.001						
mAST/ALT ratio	3.212	24.83 (3.492, 176.48)	0.001						
cAST/ALT ratio	0.811	2.521 (1.353, 3.745)	0.001						

B: regression coefficient; OR: odds ratio; 95% CI: confidence interval

### Predictive values of risk models for predicting prognosis in all patients

Base on the regression coefficients and actual values, several risk models containing age, α-HBDH, and AST/ALT or mAST/ALT or cAST/ALT ratios were constructed as follows:

Model1(M1) = 0.092×age + 0.71×AST/ALT ratio + 0.003 × α-HBDH.

The AUC of M1 was 0.8888 for the prediction of mortality. We further simplified it into simple M 1 (sM1) = 0.1×age + AST/ALT ratio + 0.003 × α-HBDH.

AUC of sM1 was 0.8889 for the prediction of mortality with sensitivity and specificity were 83.3 and 79.6, respectively at the cutoff value of > 11.36.

Similarly, M2, sM2, M3 and sM3 were constructed as follows:

M2 = 0.088×age + 2.525×mAST/ALT ratio + 0.003 × α-HBDH.

sM2 = 0.1×age + 2.5×mAST/ALT ratio + 0.003 × α-HBDH.

M3 = 0.094×age + 0.921×cAST/ALT ratio + 0.003 × α-HBDH.

sM3 = 0.1×age + cAST/ALT ratio + 0.003 × α-HBDH.

**Fig. 1:**
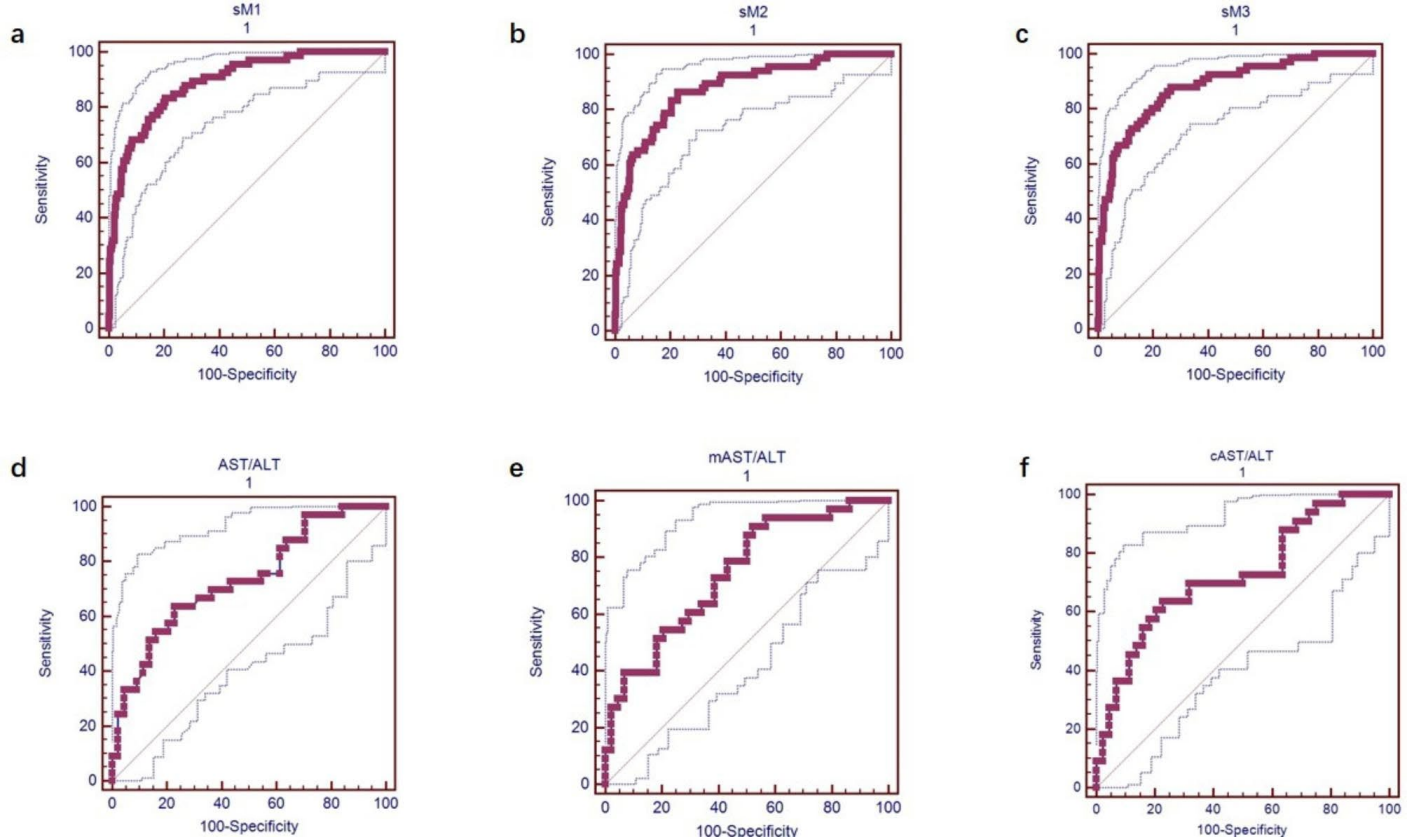
ROC of simplified risk models (sM1, sM2 and sM3) for the prediction of SFTS prognosis. (a-c) ROC curves of sM1, sM2 and sM3 for predicting SFTS mortality in all patients. (d-f) ROC curves of AST/ALT ratio, mAST/ALT ratio and cAST/ALT ratio for the prediction of SFTS mortality in ICU patients

The predictive values of the models and simplified models were comparable for mortality with similar values of AUCs, sensitivity (SEN), specificity (SPE), positive predictive value (PPV), negative predictive value (NPV), positive likelihood ratio (LR+) and negative likelihood ratio (LR-). Results are presented in Table 4; Fig. 1.

**Table 4:** Predictive efficacy of independent risk factors and constructed risk models for in-hospital mortality in all patients and ICU patients

parameters	AUC (95% CI)	cutoff value	SEN	SPE	PPV	NPV	LR+	LR-
For all patients								
Age (years)	0.674 (0.623, 0.723)	> 66	74.2	52.9	26.5	90	1.58	0.49
α-HBDH (U/L)	0.829 (0.784,0.867)	> 674.49	65.6	88.8	56.3	92.1	5.84	0.39
AST/ALT ratio	0.842 (0.800, 0.879)	> 2.26	87.9	65.1	36.5	95.9	2.51	0.19
mAST/ALT ratio	0.803 (0.757, 0.843)	> 0.659	69.2	80.6	44.6	92.1	3.57	0.38
cAST/ALT ratio	0.847 (0.805, 0.883)	> 1.728	86.2	69.2	38.6	95.7	2.8	0.2
M1	0.888 (0.851, 0.919)	> 10.21	78.8	83.4	52.0	94.5	4.74	0.25
sM1	0.889 (0.852, 0.920)	> 11.36	83.3	79.6	48.2	95.4	4.08	0.21
M2	0.878 (0.840, 0.910 )	> 9.604	83.3	81.3	50.5	95.5	4.46	0.20
sM2	0.877 (0.838, 0.909)	> 10.279	86.4	77.2	46.3	96.1	3.78	0.18
M3	0.880 (0.841, 0.912)	> 9.958	86.4	76.1	45.2	96.1	3.62	0.18
sM3	0.880 (0.842, 0.912)	> 10.562	84.9	76.5	45.2	95.7	3.61	0.20
For ICU patients								
AST/ALT ratio	0.731 (0.618, 0.826)	> 3.3	63.6	77.3	67.7	73.9	2.8	0.47
mAST/ALT ratio	0.743 (0.631, 0.836)	> 0.553	90.9	47.7	56.6	87.5	1.74	0.19
cAST/ALT ratio	0.723 (0.609, 0.819)	> 2.50	63.4	77.3	67.7	73.9	2.8	0.47

ICU: intensive care unit; AUC: area under ROC curve; SEN: sensitivity; SPE: specificity; PPV: positive predictive value; NPV: negative predictive value; LR+: positive likelihood ratio; LR-: negative likelihood ratio; M: risk score model; sM: simplified risk model; HBDH:hydroxybutyrate dehydrogenase

### Predictive values of risk factors for mortality in patients admitted to ICU

AUCs [95% confidence interval (CI)] of AST/ALT ratio, mAST/ALT ratio, and cAST/ALT ratio for the prediction of fatality were 0.731 (0.618, 0.826), 0.743 (0.631, 0.836), and 0.723 (0.609, 0.819), respectively. Of those, mAST/ALT ratio had the highest sensitivity of 90.9%.

### Survival analysis

Log-rank test of Kaplan Meier survival analysis was performed for simplified models in all patients and the risk factors in patients enrolled in ICU.

Overall survival rates of these parameters had a remarkable difference between patients with low and high values. Patients with high levels had shorter survival time and low cumulative survival rates. Risk models had higher AUCs than that of the individual risk factors included.

**Fig. 2:**
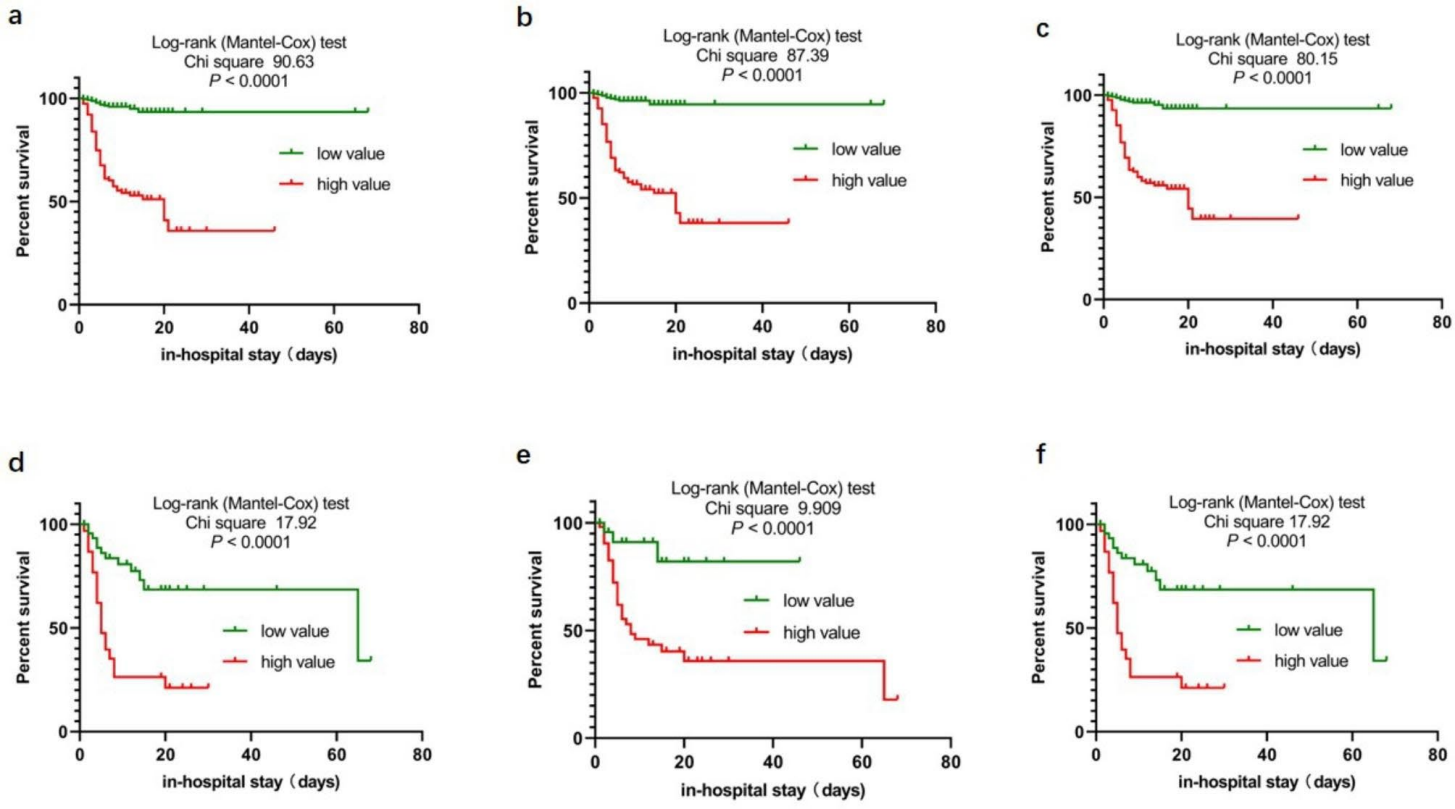
**S**urvival curves. (a-c) Survival curves of sM1, sM2 and sM3 for cumulative survival rates of all patients. (d-f) Survival curves of AST/ALT ratio, mAST/ALT ratio and cAST/ALT ratio for cumulative survival rates of ICU patients

Of these factors, high level of sM2 and mAST/ALT ratio had the highest risk of mortality with the highest hazard ratios (HRs). They were 12.68 and 5.195 in all patients and ICU patients, respectively. Results are presented in Fig. 2; Table 5.

**Table 5:** Log-rank test of Kaplan Meier survival analysis of risk models for all patients and of risk factors for ICU patients

parameters	cutoff value	HR (95% CI)	χ^2^	*P-*value
For all patients				
sM1	> 11.36	11.4 (6.751, 19.26)	90.63	< 0.0001
sM2	> 10.279	12.68 (7.599, 21.15)	87.39	< 0.0001
sM3	> 10.562	10.95 (6.574, 18.23)	80.15	< 0.0001
For ICU patients				
AST/ALT ratio	> 3.3	3.899 (1.864, 8.155)	17.92	< 0.0001
mAST/ALT ratio	> 0.553	5.195 (2.531, 10.67)	9.909	< 0.0001
cAST/ALT ratio	> 10.562	3.899 (1.864, 8.155)	17.92	< 0.0001

h: hazard ratio

## Discussion

With the increase of incidence year by year worldwide, SFTSV infection has been listed as prioritised disease for research and development by the World Health Organization (WHO) in 2018 [17]. Virus-induced histopathological lesions had been identified in multiple organs, such as the spleen, lung, kidney, and liver in SFTS [18]. Colonization of SFTSV in immune cells, especially macrophages can result in cytokine storm [18, 19] which can lead to multi-organ dysfunction or even multi-organ failure if inadequately treated [20].

Our results showed that multi-organ injury biomarkers, such as ALT and AST (hepatocellular injury), ALP and GGT (cholestatic injury), CK (muscle damage), CKMB and hsTnI (cardiac injury), and LDH and α-HBDH (multiorgan involvement) were elevated significantly in death cases than those in survivors. AST/ALT, mAST/ALT ratio and cAST/ALT ratios were independent risk factors for fatality of all SFTS patients and patients admitted to ICU. A previous study [14] demonstrated that the leakage patterns of mAST and cAST were quite different in ischemic necrosis of the rat liver: cAST reached a peak level soon after the restoration of circulation to the ischemic liver, while level of mAST increased slowly. And the activity of mAST was relatively stable compared with that of cAST and the cumulative activity of circulating mAST had a fairly well correlation with the decrease of total adenine nucleotides which were used to measure cell viability. This suggested that mAST was a useful biomarker of liver ischemic necrosis. In chronic liver injury and myocardial necrosis in humans, the release patterns of mAST were similar to that in rats [15, 21].

Our results showed that the risk models containing the respective three ratios had comparatively high predictive values for mortality in all patients which indicated that they can all be used to predict clinical outcome of SFTS. In the ICU patients, mAST/ALT ratio had the highest AUC for the prediction of adverse clinical outcome with the highest sensitivity of 90.9%. Therefore, mAST/ALT ratio can be used as an indicator for the prediction of an unfavorable outcome in patients in severe condition. It is notable that among independent risk factors, mAST/ALT ratio had the highest OR value in all patients and in patients enrolled in ICU. This indicated that with the elevation of mAST/ALT ratio, patients at a relatively higher risk of death than with elevations of other factors.

In survival analysis, sM2 containing mAST/ALT ratio had the highest HR value (12.68) among the risk models in all patients. In patients admitted to ICU, mAST/ALT ratio had the highest HR value (5.195) among three ratios. These results demonstrated that among three ratios, patients with high mAST/ALT ratio had a higher risk of mortality than with a low value compared with other ratios.

In our results, age is a risk factor for mortality in SFTS and values of three ratios had significant correlation with age. It has been reported that high AST/ALT ratio was most closely associated with all-cause mortality in the elderly [22]. This suggested that age was another factor for the elevation of three ratios.

In this study, α-HBDH is another risk factor for SFTS mortality. As known, α-HBDH is an important enzyme in glucose metabolism, which is widely distributed in various tissues and organs, especially in the heart, kidney, and brain, and is usually used to assess heart and brain damage in clinical practice [23, 24]. It has been reported that α‐HBDH was an independent risk factor for mortality in virus disease, such as COVID-19 infection [25]. It is the first time that in our study to evaluate its predictive value for clinical outcome in SFTS.

Previous study revealed that sepsis-induced myocardial dysfunction (SIMD) was associated with worse clinical outcomes, and mitochondrial dysfunction was an important aspect of SIMD development [26]. This is in accordance with our results that biomarkers of cardiac injury such as hsTnI and AST levels were dramatically elevated in death group than those in recovers.

In addition to sepsis induced by virus infection, virus infection can lead to mitochondrial dysfunction with subsequent metabolic abnormality and ATP deficiency, excessive release of reactive oxygen species (ROS), which contribute to organ failure [27]. Mitochondria damage in sepsis can result in the activation of mtDAMP-mediated signaling pathway and further damage mitochondria leading to more mtDAMP release, which forms a vicious circle. Interrupting this cycle is a hopeful strategy in the prevention and treatment of sepsis [28]. Not only acts as an aminotransferase, mAST is also a high-affinity long-chain fatty acids (LCFA) binding site which plays a key role in amino acid metabolism and transport of the LCFA affecting cell function and fate[29]. Interruption of vicious metabolism cycle holds a promising prospect for treatment of SFTS and mAST can be intervention target.

There are several limitations in this study, firstly, data of mAST in some patients with mild condition were absent resulting in a high mortality rate than the results showed in other studies. Secondly, the number of ICU patients was small which suggested that the results need further confirmation in a large cohort in the future.

In all, elevation of AST and mAST was multifactorial and mAST/ALT ratio is a great predictive factor for adverse clinical outcome in SFTS patients, especially in those in severe condition.

## Data Availability

The datasets generated and/or analysed during the current study are not publicly available due to secrecy but are available from the corresponding author on reasonable request.

## Abbreviations

ALT: Alanine aminotransferase
AST: Aspartate aminotransferase
ALP: Alkaline phosphatase
AUC: Area under ROC curve
cAST: Cytosolic AST
CK: Creatine kinase
CK-MB: CK heart-type isoenzyme
CI: Confidence interval
GGT: Gamma-glutamyltransferase
hsTnI: High-sensitivity troponin I
α-HBDH: α-hydroxybutyrate
HR: Hazard ratio
LDH: Lactate dehydrogenase
LR+: Positive likelihood ratio
LR-: Negative likelihood ratio
mAST: mitochondrial AST
M: Risk score model
NPV: Negative predictive value
OR: Odds ratio
PPV: Positive predictive value
ROC: Receiver operating characteristic curve
SEN: sensitivity
SPE: specificity
sM: simplified risk model
SFTS: Severe fever with thrombocytopenia syndrome
SFTSV: SFTS virus
